# Sexually Transmitted Infections in Male Patients with Urethritis

**DOI:** 10.3390/pathogens12121434

**Published:** 2023-12-10

**Authors:** Jorge Llaca-Díaz, Victoria Medina-Loredo, Dayra Huerta-López, Néstor Casillas-Vega

**Affiliations:** Departamento de Patología Clínica, Hospital Universitario “Dr. José Eleuterio González”, Universidad Autόnoma de Nuevo Leόn, Ave. Francisco I. Madero, Mitras Centro, Monterrey C.P. 64460, Mexico; jorge.llacadz@uanl.edu.mx (J.L.-D.); victoria.medinalo@uanl.edu.mx (V.M.-L.); dayra.huerta.9@gmail.com (D.H.-L.)

**Keywords:** sexually transmitted disease, men, *Chlamydia trachomatis*, *Ureaplasma urealyticum*, *Mycoplasma genitalium*

## Abstract

(1) Background: Sexually Transmitted Infections (STIs) in men are a significant public health problem due to the consequences they can have, such as chronic diseases, infertility, cancer, and even death. This study aimed to determine the frequency of microorganisms associated with STIs in men with urethritis attending urology consultations, and to explore their clinical correlations. (2) Methods: A population that attended the urology consultation of the University Hospital “Dr. José E. González” was studied. Written consent was obtained, and interviews and clinical history were conducted about specific risk factors identifying signs and symptoms associated with any genitourinary condition; after that, urine samples were collected. Identification of *C. trachomatis*, *N. gonorrhoeae*, *U. urealyticum*, and *M. genitalium* was based on amplifying species-specific DNA fragments. (3) Results: A total of 200 patients were included. The mean age was 55 years (20–95). According to the interviews, only 32.5% (n = 65) had received prior sex education. Additionally, 75% (n = 150) do not usually use any protection during sexual intercourse. Regarding clinical factors, 69.4% (n = 138) presented burning or pain when urinating. Molecular analysis revealed the presence of *C. trachomatis* to be 9.5% (n = 19), with *U. urealyticum* at 13% (n = 26), and *M. genitalium* at 2% (n = 4). (4) Conclusions: This is the most extensive molecular epidemiological study of the frequency of STIs in men in Mexico in third-level care and its association with different risk factors. As reported globally, a similar frequency of *C. trachomatis*, *U. urealyticum*, and *M. genitalium* was detected.

## 1. Introduction

Sexually transmitted infections (STIs) in men are a significant public health problem due to the consequences they can have, such as chronic diseases, infertility, cancer, and even death [1]. Numerous risk factors that influence the appearance of an STI have been identified in men, the most important being the number of sexual partners, previous sexual education, inconsistent condom use, and previous disease or STIs [2]. In addition, global morbidity and mortality from sexually transmitted pathogens compromise quality of life, and sexual and reproductive health [3].

STIs are the most common cause of infectious urethritis [4]. Coinfection of several agents is frequent, mainly involved in *Neisseria gonorrhoeae* and *Chlamydia trachomatis.* Other microorganisms include *Mycoplasma genitalium*, *Trichomonas vaginalis*, adenovirus, and herpes simplex [5].

Urethritis is characterized by urethral inflammation and inflammation of the periurethral glands, occurring with dysuria; in at least 50% of cases, a urethral flow originates either purulent, mucopurulent, serous, or in more severe cases, a hemorrhagic flow, as well as asymptomatic cases. The non-gonococcal origin of the infection will usually present with fewer symptoms [6].

There is little information on the causative agents of STIs in our country since there are no relevant studies on the detection of these microorganisms, and reflections on their association with sociodemographic, behavioral, and clinical factors, as well as coinfections with different pathogens associated with STIs in men in a tertiary care hospital. Therefore, the main purpose of this study was to determine the frequency of microorganisms associated with sexually transmitted infections in men with urethritis who attend the urology consultation, and their clinical correlation.

## 2. Materials and Methods

### 2.1. Study Population

Two hundred male patients attended the Urology consultation at the Hospital—“Dr. José E. González”—of the Universidad Autónoma de Nuevo Leon in Monterrey, Mexico. The study was carried out from January 2019 to June 2020. In addition, sociodemographic, behavioral, and clinical factors associated with genital infections were collected. The inclusion criteria were that the patients were older than 18 years, and that they came to the consultation for the first time. The exclusion criteria were patients who did not provide their informed consent, or made an inadequate sample collection. This study was approved by the Ethics and Research Committee of the Facultad de Medicina, Universidad Autónoma de Nuevo León in Monterrey, Mexico (approval no. PC18-00005). All patients were granted informed consent in writing or orally, as approved by the Ethics Committee. Patients who tested positive for STIs were notified, screened, and treated. All patients underwent a rapid screening test using a point-care test to detect HIV.

### 2.2. Sample Collection and DNA Extraction

For admission of the samples, urine collection was stipulated in a sterile container provided in the urology consultation to the patients, which contained boric acid for conservation; they were asked for the first jet of urination, with a volume between 10 and 20 mL. The sample was taken to the laboratory in less than 2 h. DNA was extracted using the commercial ISOLATE II Genomic DNA Kit (Bioline, London, UK).

### 2.3. PCR Detection

PCR was performed in a Biometra TOne Thermal Cycler (Analytic Jena AG, Montreal, QC H3C 0J7, Canada). The detection for *C. trachomatis* was performed by amplification of the *Phospholipase D Endonuclease Superfamily Protein* (*PLDESP*) gene [7]; for the PCR reaction, the total volume was 25 μL, consisting of 12.5 μL of Master Mix (GoTaq^®^ Green Maker Mix Protocol, Promega, Madison, WI, USA), 0.75 μL of MgCl_2_ (3 mM), 7.75 μL of sterile water, 2 μL of template DNA, and 1 μL of forward and reverse primers CT-F (5′-TCTTTTTAAACCTCCGGAACCCACTT-3′) and CT-R (5′-GGATGGCATCGCATAGCATTCTTTG-3′), respectively. The amplification conditions were: 5 min at 94 °C, 30 cycles of 1 min at 95 °C, 30 s at 57 °C, 22 s at 72 °C, and final extension at 72 °C for 7 min. The size of the PCR product was 360 bp.

*N. gonorrhoeae* was detected by amplification of the *cppB* gene [8]; for the PCR reaction, the total volume was 25 μL, consisting of 12.5 μL of Master Mix (GoTaq^®^ Green Maker Mix Protocol, Promega), 5.5 μL of sterile water, 5 μL of template DNA, and 1 μL of forward and reverse primers NG-F (5′-CGGCAGCATTCAATTTGTT-3′) and NG-R (5′-AAAAAGCCGCCATTTTTGTA-3′), respectively. The amplification conditions were: 10 min at 94 °C, 35 cycles of 1 min at 94 °C, 1 min at 62 °C, 1 min at 72 °C, and final extension at 72 °C for 10 min. The size of the PCR product was 162 bp.

*U. urealyticum* was detected by amplification of the *urease* gene [9]; for the PCR reaction, the total volume was 25 μL, consisting of 12.5 μL of Master Mix (GoTaq^®^ Green Maker Mix Protocol, Promega), 7.5 μL of sterile water, 3 μL of template DNA, and 1 μL of forward and reverse primers UU-F (5′-GGATTTGTTAGATATCGTCAAGG-3′) and UU-R (5′-TCATCTTTTAAAGCTCCACATTATTAGT-3′), respectively. The amplification conditions were: 5 min at 95 °C, 35 cycles of 20 s at 95 °C, 1 min at 62 °C, 1 min at 72 °C, and final extension at 72 °C for 5 min. The size of the PCR product was 429 bp.

*M. genitalium* was detected by amplification of the *MgPa* gene [10]; for the PCR reaction, the total volume was 25 μL, consisting of 12.5 μL of Master Mix (GoTaq^®^ Green Maker Mix Protocol, Promega), 6.5 μL of sterile water, 5 μL of template DNA, and 1 μL of forward and reverse primers MG-F (5′-ACCTTGATGGTCAGCAAAACTT-3′) and MG-R (5′-CCTTTGATCTCATTCCAATCAGTA-3′), respectively. The amplification conditions were: 5 min at 95 °C, 35 cycles of 20 s at 95 °C, 1 min at 62 °C, 1 min at 72 °C, and final extension at 72 °C for 5 min. The size of the PCR product was 281 bp.

The amplification products were visualized using electrophoresis on a 2.5% agarose gel stained with 1 μg/mL ethidium bromide, compared with a standard DNA molecular weight (DNA Molecular Weight, Marker 100, Sigma Aldrich, St. Louis, MO, USA), and developed with a UV transilluminator (UVP Transilluminator M-20V, Analytik-Jena, Beverly, MA, USA).

### 2.4. Statistical Analysis

The qualitative variables were summarized by calculating absolute frequency expressed in percentages. Multivariate logistic regression models were used to estimate the odds ratio (OR) with 95% confidence intervals (95% CI) for the association between risk factors and the prevalence of STIs. All the PCR assays were performed in duplicate to confirm the detection of the microorganism. *p*-values ≤ 0.05 were considered significant. All statistical analyses were performed using R software (V 4.2.2) (https://www.r-project.org/ (accessed on 17 October 2023)

## 3. Results

The mean age of the patients in this study was 55 years (20–95 years). Regarding schooling, 34.5% (n = 69) only completed secondary school; 32% (n = 64) primary; 15.5% (n = 31) higher education; 13% (n = 26) high school; and 5% (n = 10) had no schooling. Additionally, 67.55% (n = 65) never received any type of sex education; 51.5% (n = 103) reported having 0 to 5 lifetime sexual partners; and 96% (n = 192) reported only having heterosexual relations. Furthermore, 75% (n = 150) do not usually use any protection during sexual intercourse, and 29.5% (n = 59) said they had paid for sex. The most frequently occurring symptom was burning or painful urination at 69.4% (n = 138), followed by testicular inflammation at 11.9% (n = 23.8), penile discharge at 8.5% (n = 17), genital warts at 5.5% (n = 11), and genital blisters or sores at 3% (n = 6). No patient tested positive for HIV.

*C. trachomatis* was detected in 9.5% (n = 19) by the *PLDESP* gene, *U. urealyticum* in 13% (n = 26) by amplifying the *urease* gene, *M. genitalium* in 2% (n = 4) by amplifying the MgPa gene, and no positive isolation for *N. gonorrhoeae* was detected.

An association was identified with the different risk factors analyzed with a 95% confidence level, where the Odds Ratio variables showed that a value of >1 was considered a risk factor, while values <1 were considered risk factors. *C. trachomatis* and *M. genitalium* infections were more frequent in patients aged ≥50 years (OR = 0.09, 95% CI 0.01–10.43, and OR = 0.32, 95% CI 0.01–10.44, respectively), and *U. urealyticum* in patients aged 40–49 years (OR = 0.06, 95% CI 0.08–6.04); regarding the population that did not receive some type of sex education throughout their lives, a correlation was found with *C. trachomatis* (OR = 0.49.95% CI 0.18–7.33), *U. urealyticum* (OR = 0.67, 95% CI 0. 28–1.55), and *M. genitalium* (OR = 0.47, 95% CI 0.04–4.65); for those who had more than 20 sexual partners throughout their life, a correlation was found with *C. trachomatis* (OR = 4.01, 95% CI 15.46–41.71) and *M. genitalium* (OR = 3.46, 95% CI 0.11–3.45); and finally, a correlation of *C. trachomatis* was observed with patients who reported that they had never used a contraceptive method during sexual intercourse (OR = 1.20, 95% CI 0.25–5.70), Table 1.

## 4. Discussion

### 4.1. Prevalence Comparison

The prevalence of STIs in Mexico in the case of men is not well known, since limited studies are conducted to evaluate pathogens related to *C. trachomatis*, *N. gonorrhoeae*, *U. urealyticum*, and *M. genitalium*, so the information obtained is not representative of the male population as a whole [11].

The overall frequency of STIs observed in this study’s population was 38.7% (n = 79), which is similar to another study conducted in Spain, which included 203 samples of urine and urethral exudates in men who were in prison, in which it was detected that 40.5% of the population suffered from at least one STI [12]; it is also similar to another study conducted in Germany, in which the overall prevalence of STIs was 30.1% in male patients who had sex with men [13]. However, compared to other studies, the reported frequency was lower, with the study conducted by Rich et al. in Israel reporting a prevalence of 26.7% in heterosexual men attending the STI clinic [14]; similarly, the case of the study conducted by Kupprat and collaborators in the United States on 169 sexually active male patients where at least 35% maintained a bisexual relationship, presented an STI frequency of 50% [15]. It should be noted that the study conducted in this work was in a tertiary health care hospital, as in the previously mentioned studies. The prevalence of *C. trachomatis* has been identified in different countries. In our study, a frequency of 9.5% (n = 13) was obtained. The only previous study carried out in the Mexican male population was with urethral samples, of which only 3.6% (n = 14) presented an active infection [16]; in another study carried out in our country, 659 urine samples from men whose sexual partners were infertile women were analyzed, obtaining a prevalence of 7.4% [17].

*N. gonorrhoeae* is responsible for gonorrhea. The WHO estimates that it has an incidence rate in America of 0.6% in men aged 15 to 49 [3]. In this study, we did not detect the presence of this bacterium, which could be because the type of sample used, in this case, urine, was not as significant as another type of sample, such as a urethral exudate. It is essential to emphasize that this bacterium does not survive more than 24 h at room temperature, and that a unique transport medium is needed [18]. In this study, the sample was at 4 °C, and Amies liquid medium with flocked swabs was used.

*U. urealyticum* is an etiological agent primarily related to male urethritis and is a normal genital microbiota. A study conducted on male patients using urine samples from 83 patients obtained a frequency of 4.8% [19], much lower than that reported in this study. In contrast, in a study conducted in Mexico by Solís and collaborators, they reported a frequency of 53% for *U. urealyticum* infection [20], which is a much higher figure than reported in this study. As for the variation of the different studies, it can be attributed to the fact that *U. urealyticum* is regularly related to the microbiota of the male urethra, and that if an infection by this bacterium occurs, it is even more related to the semen sample and male infertility, according to a study by Rodríguez and collaborators, in which they detected a higher frequency than that reported in this study, with 76% [21]. The frequency of *U. urealyticum* varies in the literature from 5 to 42%, despite the contradictory results shown about its pathogenicity in studies conducted in men [22]. In another study conducted at Hiroshima University Hospital from December 2021 to May 2022, a frequency of 2.3% was detected in male urine, which is lower than the prevalence found in our study’s population [23].

*M. genitalium* is recognized as an etiologic agent of non-gonococcal urethritis in men. A study conducted on men from urine samples of patients with urethritis found a frequency of 12% [19], higher than in this study. In contrast, Gimenes and collaborators conducted a study where a prevalence of *M. genitalium* of 3% was obtained in urine samples, similar to our study. The prevalence of *M. genitalium* infections reported globally ranges from 1 to 10% [7].

### 4.2. Public Health Implications

Patients who did not receive some type of sex education throughout their lives, had sex with more than 20 people, and did not frequently use a contraceptive method were at increased risk of contracting a *C. trachomatis* infection. This can be explained because this population does not prevent infection with the use of contraceptive methods, thus confirming the studies carried out by Rondeau and collaborators, where early sex education is associated with the prevention of infection in the future, thus establishing a base knowledge so that they know the different complications that the disease has [24].

The study’s population was patients who already had a previous disease, which is urethritis, in this case, reported as non-gonococcal. Hence, it is possible to attribute that they have already received prior treatment. However, it is known that infection by *N. gonorrhoeae* can be transient and relapse, despite being treated previously [18]. *M. genitalium* is detected more frequently in populations with risk factors such as the number of sexual partners throughout their lives, reaching up to 5% in males [25].

STIs are a significant public health problem that affects both men and women. However, in this study, we focused on the implications of STIs in men. The implications of STIs can go beyond physical health issues, affecting your psychological well-being and quality of life as well. A key strategy for preventing STIs in men is promoting consistent and correct condom use. Education on the importance of protection during sexual intercourse, as well as the accessible availability of condoms, are essential elements in this preventive approach. Additionally, it is crucial to encourage open and honest communication about sexual health between men and their partners, reducing the stigma associated with STIs and promoting an environment conducive to informed decision-making. Early detection and timely treatment are of paramount importance over the health implications.

A potential limitation of the study was the specificity of the population, since it was only in male patients who came to the clinic for discomfort that, in this case, was urethritis, and it was not carried out in an open population, which could give us a greater perspective of the real frequency in the entire population, without bias. It should also be noted that this study was carried out in a tertiary care hospital, which makes the population different from that of a first-level of care, since, at the time of going to our hospital, many of the patients reported that they had previously gone to a first-level consultation, but did not see improvement with their discomfort. This opens the way for another study, but with a population without symptoms since most STIs are asymptomatic. By making a timely diagnosis, transmission and severe health complications caused by these microorganisms can be avoided.

## 5. Conclusions

This is the largest molecular epidemiological study examining the frequency of *C. trachomatis*, *N. gonorrhoeae*, *U. urealyticum*, and *M. genitalium* in men with urethritis in tertiary care in Mexico, and its association with different risk factors. A similar frequency of *C. trachomatis*, *U. urealyticum*, and *M. genitalium* was detected and reported worldwide. STIs profoundly impact the health of men with urethritis, encompassing their physical, emotional, and social well-being. Addressing this issue requires a comprehensive approach that includes promoting safe sex practices, education, early detection, and combating stigma, as the psychological impact of STIs on men is significant and multifaceted. An approach that not only focuses on the physical aspect of these diseases, but also provides emotional and psychological support to address the mental and emotional challenges faced by those affected by STIs is necessary. Prevention and effective treatment of STIs are essential to protect men’s health and prevent the spread of these infections.

## Figures and Tables

**Table 1:** Associations of sexually transmitted pathogens with sociodemographic, behavioral, and clinical characteristics of the total number of patients who attended urology consultation.

	Total *	*C. trachomatis*			*U. urealyticum*			*M. genitalium*		
Characteristics	n = 200% (n)	*p*-Value	OR	95% CI	*p*-Value	OR	95% CI	*p*-Value	OR	95% CI
**Sociodemographic Factors**										
**Age group (years)**										
20–29	11 (22)	0.256	4.71	0.74–5.30	0.350	4.71	0.77–5.40	0.256	4.71	0.74–5.30
30–39	12 (24)	0.324	4.01	0.18–1.33	0.345	4.01	0.01–2.33	0.324	4.01	0.18–1.3
40–49	9.5 (19)	0.085	0.06	0.01–7.04	0.050	0.06	0.08–6.04	0.085	0.06	0.07–7.04
≥ 50	67.5 (135)	0.045	0.09	0.01–10.43	0.032	0.09	0.01–7.48	0.035	0.32	0.01–10.44
**Scholarship**										
None	5 (10)	0.758	0.91	0.01–10.95	0.736	0.90	0.07–8.92	0.758	0.91	0.01–10.95
Elementary	32 (64)	0.513	0.22	0.01–19.61	0.421	0.22	0.02–14.13	0.513	0.22	0.01–12.98
Middle school	34.5 (69)	0.256	0.06	0.07–7.04	0.299	0.06	0.01–5.04	0.256	0.06	0.01–6.54
High school	13 (26)	0.324	0.09	0.01–10.43	0.216	0.09	0.01–9.43	0.324	0.09	0.01–8.43
Bachelor’s	15.5 (31)	0.457	0.18	0.01–16.63	0.996	0.18	0.02–16.60	0.457	0.18	0.01–16.63
**Behavioral Factors**										
**Sex education**										
Yes	32.5 (65)	0.061	2.00	0.74–5.30	0.176	1.49	0.64–3.52	0.247	2.10	0.21–2.05
No	67.5 (135)	0.041	0.49	0.18–7.33	0.020	0.67	0.28–1.55	0.043	0.47	0.04–4.65
**No. of previous sex partners**										
0–5	51.5 (103)	0.309	0.16	0.01–3.48	0.175	0.73	0.74–5.30	0.068	0.18	0.11–2.34
6–10	21.5 (43)	0.087	7.41	0.78–70.22	0.270	0.49	0.18–1.33	0.119	0.19	0.22–1.20
11–20	11.5 (23)	0.071	4.71	0.23–9.11	0.165	0.27	0.51–3.69	0.981	1.05	0.62–3.98
≥20	14 (28)	0.034	4.01	15.46–41.71	0.736	2.01	0.67–7.61	0.050	3.46	0.11–3.45
**Contraceptive methods (frequency)**										
Yes	9 (18)	0.919	0.83	0.02–24.45	0.954	0.88	0.02–6.60	0.176	1.49	0.64–3.52
Most of the time	16 (32)	0.130	0.08	0.01–2.01	0.131	0.08	0.01–2.6	0.186	3.17	0.28–1.55
No	75 (150)	0.015	1.20	0.25–5.70	0.825	1.25	0.37–6.80	0.175	0.31	0.11–3.08
**Payment for sex**										
Yes	29.5 (59)	0.765	0.73	0.09–5.52	0.147	1.59	0.65–3.75	0.452	0.79	0.02–7.61
No	70.5 (141)	0.388	1.19	0.25–2.39	0.134	0.61	0.26–1.53	0.135	1.25	0.13–3.37
**Sexual relations under the influence of alcohol and/or drugs**										
Yes	24 (48)	0.971	0.96	0.10–8.92	0.077	0.94	0.32–2.44	0.062	0.36	0.029–2.73
No	76 (152)	0.087	1.94	0.72–4.91	0.467	1.06	0.40–3.05	0.014	0.11	0.01–2.44
**Clinical factors**										
**Symptoms**										
Burning urination	69.4 (138)	0.524	0.24	0.01–31.29	0.565	0.24	0.01–31.29	0.430	0.26	0.02–3.16
Testicular inflammation	11.9 (23)	0.401	1.15	0.25–3.94	0.997	2.56	0.40–46.73	0.982	1.29	0.38–7.94
Penile discharge	8.5 (17)	0.335	0.57	0.24–3.48	0.997	2.92	0.24–6.38	0.915	0.99	0.33–5.67
Genital warts	5.5 (11)	0.536	0.44	0.03–5.91	0.335	0.44	0.03–5.91	0.760	0.23	0.03–5.92
Blisters or sores	3 (6)	0.668	0.65	0.09–4.52	0.535	0.65	0.09–4.52	0.091	0.78	0.09–3.99

* Total number of patients who attended urology consultation.

## Data Availability

Data are contained within the article.

## References

[1] Workowski K.A. (2015). Centers for Disease Control and Prevention Sexually Transmitted Diseases Treatment Guidelines. Clin. Infect. Dis..

[2] Kreisel K.M., Spicknall I.H., Gargano J.W., Lewis F.M., Lewis R.M., Markowitz L.E., Roberts H., Johnson J.S., Song R., St. Cyr S.B. (2021). Sexually Transmitted Infections Among US Women and Men: Prevalence and Incidence Estimates, 2018. Sex. Transm. Dis..

[3] World Health Organization (2019). Progress Report on HIV, Viral Hepatitis and Sexually Transmitted Infections 2019: Accountability for the Global Health Sector Strategies, 2016–2021 (No. WHO/CDS/HIV/19.7).

[4] Brill J.R. (2010). Diagnosis and treatment of urethritis in men. Am. Fam. Physician.

[5] De Souza L.S., Sardinha J.C., Talhari S., Heibel M., Santos M.N.D., Talhari C. (2021). Main etiological agents identified in 170 men with urethritis attended at the Fundação Alfredo da Matta, Manaus, Amazonas, Brazil. An. Bras. Dermatol..

[6] Leos-Alvarado C., Llaca-Díaz J., Flores-Aréchiga A., Pérez-Chávez F., Casillas-Vega N. (2020). Male urethritis. A review of the ideal diagnostic method. Uretritis masculina. Una revisión del método ideal de diagnóstico. Actas Urol. Esp..

[7] Gimenes F., Medina F.S., Abreu A.L.P.D., Irie M.M.T., Esquicati I.B., Malagutti N., Vasconcellos V.R.B., Discacciati M.G., Bonini M.G., Maria-Engler S.S. (2014). Sensitive simultaneous detection of seven sexually transmitted agents in semen by multiplex-PCR and of HPV by single PCR. PLoS ONE.

[8] Martínez M.A. (2009). Diagnóstico microbiológico de las infecciones de transmisión sexual (ITS): Parte 1. ITS no virales [Microbiological diagnosis of sexually transmitted infections (STI): Part 1. Non-viral STI]. Rev. Chil. Infectol..

[9] Xiao L., Glass J.I., Paralanov V., Yooseph S., Cassell G.H., Duffy L.B., Waites K.B. (2010). Detection and characterization of human *Ureaplasma* species and serovars by real-time PCR. J. Clin. Microbiol..

[10] Jensen J.S., Uldum S.A., Søndergård-Andersen J., Vuust J., Lind K. (1991). Polymerase chain reaction for detection of Mycoplasma genitalium in clinical samples. J. Clin. Microbiol..

[11] López de Munain J. (2019). Epidemiology and current control of sexually transmitted infections. The role of STI clinics. Enferm. Infecc. Microbiol. Clin..

[12] Sánchez Recio R., Alonso Pérez de Ágreda J.P., Santabárbara Serrano J. (2016). Infecciones de transmisión sexual en hombres internos en prisión: Riesgo de desarrollo de nuevas infecciones [Sexually transmitted infections in male prison inmates: Risk of development of new diseases]. Gac. Sanit..

[13] Jansen K., Steffen G., Potthoff A., Schuppe A.K., Beer D., Jessen H., Scholten S., Spornraft-Ragaller P., Bremer V., Tiemann C. (2020). STI in times of PrEP: High prevalence of chlamydia, gonorrhea, and mycoplasma at different anatomic sites in men who have sex with men in Germany. BMC Infect. Dis..

[14] Rich R., Leventhal A., Sheffer R., Mor Z. (2018). Heterosexual men who purchase sex and attended an STI clinic in Israel: Characteristics and sexual behavior. Isr. J. Health Policy Res..

[15] Kupprat S.A., Krause K.D., Ompad D.C., Halkitis P.N. (2017). Substance Use and Cognitive Function as Drivers of Condomless Anal Sex Among HIV-Positive Gay, Bisexual, and Other Men Who Have Sex with Men Aged 50 and Older: The Gold Studies. LGBT Health.

[16] Guerra-Infante F.M., Tapia-Yáñez J.R., López-Hurtado M., Flores-Medina S., Díaz-García F.J. (2005). Infección por Chlamydia trachomatis en varones y su asociación con las alteraciones ginecológicas de su compañera sexual [*Chlamydia trachomatis* infection in men and its association with gynecologic alterations in their sexual partners]. Rev. Investig. Clin..

[17] López-Hurtado M., Escarcega-Tame M.A., Escobedo-Guerra M.R., de Haro-Cruz M.J., Guerra-Infante F.M. (2022). Identification of *Chlamydia trachomatis* genotypes in Mexican men with infertile women as sexual partners. Enferm. Infecc. Microbiol. Clin..

[18] Serra-Pladevall J., Gulin Blanco C., Vila Olmo N., Arjona Camacho P., Andreu Domingo A. (2018). Preservation of *Neisseria gonorrhoeae*: Should swabs be refrigerated or not?: Neisseria gonorrhoeae preservation. J. Microbiol. Methods.

[19] Khatib N., Bradbury C., Chalker V., Koh G.C.K.W., Smit E., Wilson S., Watson J. (2015). Prevalence of *Trichomonas vaginalis*, *Mycoplasma genitalium* and *Ureaplasma urealyticum* in men with urethritis attending an urban sexual health clinic. Int. J. STD AIDS.

[20] Martínez R.S., Castillo T.V., Celis S., Callejas L.H. (2006). Susceptibilidad de *Mycoplasma hominis* y *Ureaplasma urealyticum* ante diferentes antibióticos. Rev. Médica De La Univ. Veracruzana.

[21] Rodríguez A.G., Cortés C.G., Forero L.Y.P. (2015). Estudio retrospectivo en el diagnóstico de *Mycoplasma* y *Ureaplasma* en muestra seminal de 89 pacientes en la Ciudad de México. Rev. De La Fac. De Med. UNAM.

[22] Pendás B.V.R., Rodríguez C.O., Pérez F.S., Alonso E.D., Guerra B.N. (2013). *Micoplasma hominis*, *Ureaplasma urealyticum* y bacterias aeróbicas en el semen de hombres que consultan por infertilidad. Rev. Cuba. De Endocrinol..

[23] Tadera K., Kitagawa H., Kitano H., Hara T., Kashiyama S., Nomura T., Omori K., Shigemoto N., Yokozaki M., Ohge H. (2023). Prevalence of *Mycoplasma hominis*, *Ureaplasma urealyticum*, and *Ureaplasma parvum* detection in urine and respiratory tract samples in Hiroshima, Japan. Heliyon.

[24] Rondeau P., Valin N., Decré D., Girard P.M., Lacombe K., Surgers L. (2019). Chlamydia trachomatis screening in urine among asymptomatic men attending an STI clinic in Paris: A cross-sectional study. BMC Infect. Dis..

[25] Napierala Mavedzenge S., Weiss H.A. (2009). Association of *Mycoplasma genitalium* and HIV infection: A systematic review and meta-analysis. AIDS.

